# Supplementary material for: Clinical effectiveness of screening for age-related macular degeneration: A systematic review
Source: PLoS One. 2023 Nov 16;18(11):e0294398. doi: 10.1371/journal.pone.0294398 (PMC10653496; doi:10.1371/journal.pone.0294398)
Supplement: S1 File — (DOCX) [file pone.0294398.s002.docx]

**Supplementary material**

**S1 Table**. Final search strategy conducted on 03/29/2022.

| **Database** | **Strategy** | **Records** |
| --- | --- | --- |
| PubMed | ((((("Macular Degeneration"[Mesh] OR "Macular Degeneration" OR "Degeneration, Macular" OR "Macular Degenerations" OR "Maculopathy" OR "Maculopathies" OR "Macular Dystrophy" OR "Dystrophy, Macular" OR "Macular Dystrophies" OR "Age-Related Macular Degeneration" OR "Age Related Macular Degeneration" OR "Age-Related Macular Degenerations" OR "Macular Degeneration, Age-Related" OR "Macular Degeneration, Age Related" OR "Maculopathies, Age-Related" OR "Maculopathy, Age-Related" OR "Maculopathy, Age Related" OR "Age-Related Maculopathies" OR "Age Related Maculopathies" OR "Age-Related Maculopathy" OR "Age Related Maculopathy") OR ("Wet Macular Degeneration"[Mesh] OR "Wet Macular Degeneration" OR "Macular Degeneration, Wet")) OR ("Wet Macular Degeneration"[Mesh] OR "Wet Macular Degeneration" OR "Macular Degeneration, Wet")) OR ("Geographic Atrophy"[Mesh] OR "Geographic Atrophy" OR "Geographic Atrophies" OR "Dry Macular Degeneration" OR "Macular Degeneration, Dry")) OR ("Macular Degeneration, Age-Related, 2" [Supplementary Concept] OR "Macular Degeneration, Age-Related, 2" OR "Armd2" OR "Macular Degeneration, Senile")) AND ((("Mass Screening"[Mesh] OR "Mass Screening" OR "Mass Screenings" OR "Screening, Mass" OR "Screenings, Mass" OR "Screening" OR "Screenings") OR ("Vision Screening"[Mesh] OR "Vision Screening" OR "Screening, Vision" OR "Screenings, Vision" OR "Vision Screenings")) OR ("opportunistic screening")) | 1,297 |
| EMBASE | #1 'macular degeneration'/exp OR 'atrophia maculae luteae' OR 'atrophy, macula lutea' OR 'bilateral macular degeneration' OR 'degeneratio maculae luteae retinae' OR 'degeneration, bilateral macula' OR 'degeneration, heredomacular' OR 'degeneration, macula lutea' OR 'degeneration, retina macula' OR 'disciform macular degeneration' OR 'disease, junius kuhnt' OR 'heredomacular degeneration' OR 'junius kuhnt disease' OR 'macula atrophy' OR 'macula bilateral degeneration' OR 'macular degeneration' OR 'macula lutea atrophy' OR 'macula lutea degeneration' OR 'macula lutea degeneration, disciform' OR 'macula lutea disciform degeneration' OR 'macular atrophy' OR 'macular degeneration' OR 'macular degeneration, disciform' OR 'macular disciform degeneration' OR 'macular dystrophy' OR 'retina atrophy, macula' OR 'retina atrophy, macula lutea' OR 'retina degeneration, bilateral macula' OR 'retina degeneration, macula' OR 'retina degeneration, macula lutea' OR 'retina heredomacular degeneration' OR 'retina macula atrophy' OR 'retina macula bilateral degeneration' OR 'retina macular degeneration' OR 'retina macular degeneration, disciform' OR 'retina macula disciform degeneration' OR 'retina macula lutea atrophy'  #2 'wet macular degeneration'/exp OR 'exudative macular degeneration' OR 'neovascular age-related macular degeneration' OR 'neovascular macular degeneration' OR 'wet macular degeneration'  #3 'age related macular degeneration'/exp OR 'age related macular degeneration' OR 'age related macular degeneration' OR 'atrophy, senile central choroidal' OR 'central areolar choroidal atrophy' OR 'central areolar choroidal sclerosis' OR 'central guttate choroidal atrophy' OR 'choroid central guttate atrophy' OR 'choroid, senile central atrophy' OR 'choroidal atrophy, central areolar' OR 'choroidal atrophy, central guttate' OR 'choroidal sclerosis, central areolar' OR 'choroiditis, tays' OR 'degeneration, macula senile' OR 'degeneration, senile macula lutea' OR 'macular degeneration, senile' OR 'macula lutea senile atrophy' OR 'macula lutea senile degeneration' OR 'macula senile degeneration' OR 'macula senile disciform degeneration' OR 'macular senile atrophy' OR 'macular senile degeneration' OR 'macular senile disciform degeneration' OR 'retina degeneration, senile macular' OR 'retina macula age related degeneration' OR 'retina macula senile degeneration' OR 'retina macula senile disciform degeneration' OR 'retina senile macular degeneration' OR 'retina senile macula lutea degeneration' OR 'retina senile macular degeneration' OR 'senile atrophy, macula lutea' OR 'senile central choroidal atrophy' OR 'senile degeneration, retina macula' OR 'senile disciform macular degeneration' OR 'senile macular degeneration' OR 'senile macula hole' OR 'senile macula lutea degeneration' OR 'senile macular atrophy' OR 'senile macular degeneration' OR 'tay choroiditis'  #4 'geographic atrophy'/exp OR 'dry macular degeneration' OR 'geographic atrophy'  #5 #1 OR #2 OR #3 OR #4  #6 'screening'/exp OR 'multiple screening' OR 'prescreening' OR 'project, screening' OR 'screening' OR 'screening method' OR 'screening procedure' OR 'screening program' OR 'screening programme' OR 'screening project'  #7 'mass screening'/exp OR 'health screening' OR 'health screening program' OR 'health screening programme' OR 'longitudinal health screening program' OR 'longitudinal health screening programme' OR 'mass screening' OR 'population screening' OR 'screening, mass'  #8 'vision test'/exp OR 'test, vision' OR 'vision screening' OR 'vision test' OR 'vision testing' OR 'vision tests' OR 'visual testing'  #9 'opportunistic screening'/exp  #10 #6 OR #7 OR #8 OR #9  #11 #5 AND #10  #12 [embase]/lim NOT ([embase]/lim AND [medline]/lim)  #13 #11 AND #12 | 3,632 |
| **Cochrane Library** | #1 MeSH descriptor: [Macular Degeneration] explode all trees 2734  #2 ("macular degeneration") (Word variations have been searched) 3589  #3 ("age related macular degeneration") 3143  #4 MeSH descriptor: [Geographic Atrophy] explode all trees 151  #5 ("geographic atrophy") 444  #6 ("Dry Macular Degeneration") 8  #7 MeSH descriptor: [Wet Macular Degeneration] explode all trees 406  #8 ("Wet Macular Degeneration") 670  #9 MeSH descriptor: [Mass Screening] explode all trees 4058  #10 ("mass screening") 4218  #11 MeSH descriptor: [Vision Screening] explode all trees 97  #12 ("vision screening") 232  #13 ("screening study") 661  #14 ("screening") 67729  #15 #1 OR #2 OR #3 OR #4 #5 OR #6 OR #7 #8 4779  #16 #9 OR #10 OR #11 OR #12 OR #13 OR #14 68103  #17 #15 AND #16 275  #18 MeSH descriptor: [Macular Degeneration] explode all trees 2734  #19 ("macular degeneration") (Word variations have been searched) 3589  #20 ("age related macular degeneration") 3143  #21 MeSH descriptor: [Geographic Atrophy] explode all trees 151  #22 ("geographic atrophy") 444  #23 ("Dry Macular Degeneration") 8  #24 MeSH descriptor: [Wet Macular Degeneration] explode all trees 406  #25 ("Wet Macular Degeneration") 670  #26 MeSH descriptor: [Mass Screening] explode all trees 4058  #27 ("mass screening") 4218  #28 MeSH descriptor: [Vision Screening] explode all trees 97  #29 ("vision screening") 232  #30 ("screening study") 661  #31 ("screening") 67729  #32 ("opportunistic screening") 162  #33 #18 OR #19 OR #20 OR #21 #22 OR #23 OR #24 #25 4779  #34 #26 OR #27 OR #28 OR #29 OR #30 OR #31 OR #32 68103  #35 #33 AND #34 275 | 275 |
| LILACS | (“macular degeneration” OR “maculopathy” OR “macular dystrophy” OR “age-related macular degeneration” OR “wet macular degeneration” OR “geographic atrophy” OR “dry macular degeneration”) AND (“screening” OR “mass screening” OR “vision screening” OR “'opportunistic screening”) AND ( db:("LILACS")) | 86 |

**S2 Table**. Studies excluded after title and abstract evaluation.

| **Nº** | **Study** | **Reason for exclusion** |
| --- | --- | --- |
| 1 | AGRAWAL, Rupesh V. et al. Hand-held Ophthalmic Camera System for Early Detection and Mass Screening of Eye Diseases: A Flagship Project of the Distributed Diagnostic Home Healthcare (D2H2) Strategic Research Initiative. Investigative Ophthalmology & Visual Science, v. 53, n. 14, p. 3094-3094, 2012. | wrong population |
| 2 | Allaert, F.-A. Importance of screening in the changes of central vision by the Amsler grid. **Angeiologie**, v. 57, n. 2, p. 64-67, 2005. | wrong publication type |
| 3 | Arden, G B; J E Wolf. Colour vision testing as an aid to diagnosis and management of age related maculopathy. **The British Journal of Ophthalmology**, v. 88, n. 9, p. 1180-1185, 2004. | wrong study design |
| 4 | Ariyasu, R G; Lee, P P; Linton, K P; LaBree, L D; Azen, S P; Siu, A L. Sensitivity, specificity, and predictive values of screening tests for eye conditions in a clinic-based population. **Ophthalmology**, v. 103, n. 11, p. 1751-1760, 1996. | wrong population |
| 5 | Ausayakhun, S; Snyder, B M; Ausayakhun, S; Nanegrungsunk, O; Apivatthakakul, A; Narongchai, C; Melo, J S; Keenan, J D. Clinic-Based Eye Disease Screening Using Non-Expert Fundus Photo Graders at the Point of Screening: Diagnostic Validity and Yield. **American Journal of Ophthalmology**, v. 227, p. 245-253, 2021. | wrong study design |
| 6 | Bartlett, H; Davies, L N; Eperjesi, F. The macular mapping test: a reliability study. **BMC Ophthalmology**, v. 5, p. 18, 2005. | wrong study design |
| 7 | Burlina, P; Freund, D E; Dupas, B; Bressler, N. Automatic screening of age-related macular degeneration and retinal abnormalities. **33rd Annual International Conference of the IEEE Engineering in Medicine and Biology Society**, p. 3962-3966, 2011. | wrong population |
| 8 | Calcagni, A; Howells, O; Bartlett, H; Denniston, A K O; Gibson, J M; Hogg, C R; Matthews, T D; Eperjesi, F. Comparison of colour contrast sensitivity in eyes at high risk of neovascular age‐related macular degeneration with and without subsequent choroidal neovascular membrane development. **Eye Eye (London, England)**, 2022. | wrong population |
| 9 | Chen, L.-S.; Tsai, C.-Y.; Liu, T.-Y.; Tung, T.-H.; Chiu, Y.-H.; Chan, C.-C.; Liou, D.-M.; Chen, T.H.-H. Feasibility of tele-ophthalmology for screening for eye disease in remote communities. **Journal of Telemedicine and Telecare**, v. 10, n. 6, p. 337-341, 2004. | wrong study design |
| 10 | Chew, E Y; Clemons, T E; Bressler, S B; Elman, M J; Danis, R P; Domalpally, A; Heier, J S; Kim, J E; Garfinkel, R A. Randomized trial of the ForeseeHome monitoring device for early detection of neovascular age-related macular degeneration. The HOme Monitoring of the Eye (HOME) study design - HOME Study report number 1. **Contemporary Clinical Trials**, v. 37, n. 2, p. 294-300, 2014. | wrong population |
| 11 | De Bats, F; Nitenberg, C V; Fantino, B; Kodjikian, L. DODMLA: Organized screening of age-related macular degeneration by delayedreading of retinophotography. **Investigative Ophthalmology and Visual Science**, v. 54, n. 15, p. 251, 2013. | wrong publication type |
| 12 | Duchin, K S; Asefzadeh, B; Poulaki, V; Rett, D; Marescalchi, P; Cavallerano, A. Teleretinal imaging for detection of referable macular degeneration. **Optometry and Vision Science**, v. 92, n. 6, p. 714-718, 2015. | wrong population |
| 13 | Hadziahmetovic, M; Mettu, P S; Cousins, S W. Remote diagnosis of referable macular pathology in high disease prevalence communities. **Investigative Ophthalmology and Visual Science**, v. 60, n. 9, p. 6126, 2019. | wrong study design |
| 14 | Han, D P. The foreseehome device and the home study: A milestone in the self-detection of neovascular age-related macular degeneration. **JAMA Ophthalmology**, v. 132, n. 10, p. 1167-1168, 2014. | wrong study design |
| 15 | Jackson, G R; Scott, I U; Kim, I K; Quillen, D A; Iannaccone, A; Edwards, J G. Author response: Additional considerations in the utility of dark adaptometry for the diagnosis of age-related macular degeneration. **Investigative Ophthalmology and Visual Science**, v. 55, n. 5, p. 3149, 2014. | wrong study design |
| 16 | Jain, S. Screening for suspicious macular lesions using a telemedicine link. **Eye**, v. 21, n. 2, p. 302, 2007. | wrong study design |
| 17 | Jain, S; Hamada, S; Membrey, W L; Chong, V. Screening for age-related macular degeneration using nonstereo digital fundus photographs. **Eye**, v. 20, n. 4, p. 471-475, 2006. | wrong study design |
| 18 | Jessa, Z; Evans, B J; Thomson, D W. The development & evaluation of two vision screening tools for correctable visual loss in older people. **Ophthalmic & Physiological Optics**, v. 32, n. 4, p. 332-348, 2012. | Wrong population |
| 19 | Jung, S; Coleman, A; Weintraub, N T. Vision Screening in the Elderly. **Journal of the American Medical Directors Association**, v. 8, n. 6, p. 355-362, 2007. | wrong study design |
| 20 | Kankanahalli, S; Burlina, P M; Wolfson, Y; Freund, D E; Bressler, N M. Automated classification of severity of age-related macular degeneration from fundus photographs. **Investigative Ophthalmology and Visual Science**, v. 54, n. 3, p. 1789-1796, 2013. | wrong population |
| 21 | Katibeh, M; Sabbaghi, H; Kalantarion, M; Nikkhah, H; Mousavi, B; Beiranvand, R; Ahmadieh, H; Kallestrup, P. Eye Care Utilization in A Community-oriented Mobile Screening Programme for Improving Eye Health in Iran: A Cluster Randomized Trial. **Ophthalmic Epidemiology**, v. 27, n. 6, p. 417-428, 2020. | wrong study design |
| 22 | Kroenke, K. Telemedicine screening for eye disease. **JAMA - Journal of the American Medical Association**, v. 313, n. 16, p. 1666-1667, 2015. | wrong study design |
| 23 | Le Tien, V; Strého, M; d'Athis, P; Taillandier-Heriche, E; Paillaud, E; Mahiddine, H; Coscas, G; Lejonc, J L; Soubrane, G; Souied, E H. Interobserver and intraobserver reliability of detecting age-related macular degeneration using a nonmydriatic digital camera. **American Journal of Ophthalmology**, v. 146, n. 4, p. 520-526, 2008. | wrong study design |
| 24 | Lee, P. Visual Acuity Screening Among Asymptomatic Older Adults. **JAMA**, v. 315, n. 9, p. 875-876, 2016. | wrong study design |
| 25 | LI, B et al. Improving the detection of retinal pathologies: A comparison of novel ultra-widefield imaging to standard fundus imaging. **Annals of the Academy of Medicine Singapore** - Volume 45, Issue 9, pp. S259-S259 - published 2016-01-01 | wrong publication type |
| 26 | LI, Bo et al. Prospective evaluation of teleophthalmology in screening and recurrence monitoring of neovascular age-related macular degeneration: a randomized clinical trial. **JAMA ophthalmology**, v. 133, n. 3, p. 276-282, 2015. | wrong population |
| 27 | LI, Bo et al. Prospective evaluation of Tele-Ophthalmology in initial screening and recurrence monitoring for wet Age-related Macular Degeneration (AMD). **Investigative Ophthalmology & Visual Science**, v. 55, n. 13, p. 4941-4941, 2014. | wrong publication type |
| 28 | LIM, Jennifer I. et al. Comparison of nonmydriatic digitized video fundus images with standard 35-mm slides to screen for and identify specific lesions of age-related macular degeneration. **Retina**, v. 22, n. 1, p. 59-64, 2002. | wrong population |
| 29 | LIN, Kenneth W.; HOLLIS, Ewell M. Screening for impaired visual acuity in older adults. **American family physician**, v. 83, n. 2, p. 189, 2011. | wrong publication type |
| 30 | MAA, April Y. et al. A novel tele-eye protocol for ocular disease detection and access to eye care services. **Telemedicine and e-Health**, v. 20, n. 4, p. 318-323, 2014. | wrong publication type |
| 31 | MAA, April Y. et al. Diagnostic accuracy of technology-based eye care services: the technology-based eye care services compare trial part I. Ophthalmology, v. 127, n. 1, p. 38-44, 2020. | wrong population |
| 32 | MAA, April Y. et al. Early experience with technology-based eye care services (TECS): a novel ophthalmologic telemedicine initiative. Ophthalmology, v. 124, n. 4, p. 539-546, 2017. | wrong population |
| 33 | MARUYAMA-INOUE, Maiko et al. Sensitivity and specificity of high-resolution wide field fundus imaging for detecting neovascular age-related macular degeneration. **PLoS One**, v. 15, n. 8, p. e0238072, 2020. | wrong population |
| 34 | MOOKIAH, Muthu Rama Krishnan et al. Automated diagnosis of age-related macular degeneration using greyscale features from digital fundus images. **Computers in biology and medicine**, v. 53, p. 55-64, 2014. | wrong publication type |
| 35 | O'BRIEN, Kieran S. et al. Cluster-randomised trial of community-based screening for eye disease in adults in Nepal: the Village-Integrated Eye Worker Trial II (VIEW II) trial protocol. **BMJ open**, v. 10, n. 10, p. e040219, 2020. | wrong publication type |
| 36 | PIRBHAI, Adnan; SHEIDOW, Thomas; HOOPER, Phil. Prospective evaluation of digital non-stereo color fundus photography as a screening tool in age-related macular degeneration. American journal of ophthalmology, v. 139, n. 3, p. 455-461, 2005. | wrong study design |
| 37 | SALTI, H.; KHOURY, J. An evaluation of photographic screening for neovascular age-related macular degeneration. **Eye**, v. 20, n. 12, p. 1374-1375, 2006. | wrong publication type |
| 38 | SHEKHAWAT, Nakul S. et al. The utility of routine fundus photography screening for posterior segment disease: a stepped-wedge, cluster-randomized trial in South India. **Ophthalmology**, v. 128, n. 7, p. 1060-1069, 2021. | wrong population |
| 39 | SILVESTER, Alexander J. Vision screening in a primary care setting. **British Journal of Ophthalmology**, 2010. | wrong publication type |
| 40 | SIU, Albert L. et al. Screening for impaired visual acuity in older adults: US preventive services task force recommendation statement. **JAMA**, v. 315, n. 9, p. 908-914, 2016. | wrong publication type |
| 41 | SUN, Qisi et al. Evaluating the Diagnostic Ability of Vision Screenings Compared to Comprehensive Eye Exams in Detecting Early Pathology. **Investigative Ophthalmology & Visual Science**, v. 58, n. 8, p. 5064-5064, 2017. | wrong publication type |
| 42 | SYNEK, S. et al. Microperimetry in the wet form of age-related macular degeneration (ARMD). **Ceska a Slovenska Oftalmologie: Casopis Ceske Oftalmologicke Spolecnosti a Slovenske Oftalmologicke Spolecnosti**, v. 70, n. 1, p. 15-20, 2014. | wrong publication type |
| 43 | SZIRTH, Ben; OOMS, Ashley; KHOURI, Albert S. Integration of OCTA in Community-Based Screening Programs for Vision-Threatening Diseases. **Investigative Ophthalmology & Visual Science**, v. 60, n. 9, p. 3039-3039, 2019. | wrong publication type |
| 44 | U.S. Preventive Services Task Force. Screening for impaired visual acuity in older adults: Recommendation statement. **American Family Physician**, v. 93, n. 12, p. 1024A-1024C, 2016. | wrong study design |
| 45 | U.S. Preventive Services Task Force. Screening older adults for eyesight problems: U.S. Preventive Services Task Force recommendation. **Annals of Internal Medicine**, v. 151, n. 1, p. 1-34, 2009. | wrong study design |
| 46 | Village-Integrated Eye Worker Trial II. 2018. Clinical trials. NCT03752840 | wrong publication type |
| 47 | WINTHER, Christina; FRISÉN, Lars. A compact rarebit test for macular diseases. **British journal of ophthalmology**, v. 94, n. 3, p. 324-327, 2010. | wrong population |
| 48 | WONG, Damon et al. An automated system for the detection of AMD-related drusen in retinal fundus images. **Investigative Ophthalmology & Visual Science**, v. 54, n. 15, p. 5494-5494, 2013. | wrong publication type |
| 49 | WOODS, Russell L.; TREGEAR, Stephen J.; MITCHELL, Reginald A. Screening for ophthalmic disease in older subjects using visual acuity and contrast sensitivity. **Ophthalmology**, v. 105, n. 12, p. 2318-2326, 1998. | wrong population |

**S3 Table**. Quality of evidence assessment using the GRADE tool.

| **Certainty assessment** | | | | | | | **Impact** | **Certainty** | **Importance** |
| --- | --- | --- | --- | --- | --- | --- | --- | --- | --- |
| **№ of studies** | **Study design** | **Risk of bias** | **Inconsistency** | **Indirectness** | **Imprecision** | **Other considerations** |  |  |  |
| **Case detection rate** | | | | | | | | | |
| 2 | observational studies | extremely serious^a,b^ | serious^c^ | not serious | not serious | none | **Midena, 2020:** CFP 7.4% (617 images); OCT 10,4% (1615 images). **Hadziahmetovic, 2019:** CFP 10.7% (34 olhos); OCT 18.9% (60 eyes); CFP and OCT 19.2% (61 eyes). | ⨁◯◯◯ Very low | CRITICAL |
| **Diagnostic accuract (sensitivity and specificity)** | | | | | | | | | |
| 2 | observational studies | very serious^b^ | very serious^c,d^ | not serious | not serious | none | **Fidalgo, 2019:** SD-OCT (total retinal or GCC thickness) - Sensitivity: 52.1% / Specificity: 67.4%; SD-OCT (total retinal thickness) - Sensitivity: 50.0% / Specificity: 73.4%; SD-OCT (GCC thickness) - Sensitivity: 37.5% / Specificity: 85.9%; SD-OCT (peripapilar RNFL thickness) - Sensitivity: 25.0% / Specificity: 93.0%. **Hadziahmetovic, 2019:** CFP - Sensitivity: 94% (95%CI, 84%-98%) / Specificity: 63% (95%CI, 53%-71%); OCT - Sensitivity: 94% (95%CI, 84%-98%) / Specificity: 93% (95%CI, 87%-96%). | ⨁◯◯◯ Very low | CRITICAL |

**CI:** confidence interval

**Explanations**

a. The study by Midena, 2020 exhibited critical risk of bias due to confounding.

b. The study by Hadziahmetovic, 2019 showed serious risk of bias due to confounding, deviations from intended interventions and missing data.

c. Individual estimates varied considerably between studies.

d. The confidence intervals (CIs) displayed minimal or no overlap.
