# Supplementary material for: Clinical effectiveness of screening for age-related macular degeneration: A systematic review
Source: PLoS One. 2023 Nov 16;18(11):e0294398. doi: 10.1371/journal.pone.0294398 (PMC10653496; doi:10.1371/journal.pone.0294398)
Supplement: Attachment — Submitted filename: Response to reviewers.docx [file pone.0294398.s004.docx]

Dear Editor,

We thank you for the opportunity to have our manuscript **PONE-D-23-03440**, entitled "Clinical effectiveness of screening for age-related macular degeneration: a systematic review", evaluated for publication by PLOS ONE. We are grateful to the reviewers for their time and effort. We are happy to implement the suggestions to improve the manuscript, as described below.

| **Questions** | **Reviewer 1** | **Reviewer 3** |
| --- | --- | --- |
| If the authors have adequately addressed your comments raised in a previous round of review and you feel that this manuscript is now acceptable for publication. | No Response | All comments have been addressed |
| Is the manuscript technically sound, and do the data support the conclusions? | Yes | Yes |
| Has the statistical analysis been performed appropriately and rigorously? | N/A | Yes |
| Have the authors made all data underlying the findings in their manuscript fully available? | Yes | Yes |
| Is the manuscript presented in an intelligible fashion and written in standard English? | Yes | No |
| Do you want your identity to be public for this peer review? | Nicholas Beare | No |

Yours sincerely,

The Authors

**Reviewer #1: “**Overall I think the authors have addressed my concerns and improved the review substantially. My main remaining concern is by the addition of information about AREDS supplementation they have failed to present the controversy regarding whether AREDS supplementation is of any benefit in AMD (see below).”

**Reviewer #1, comment 1: “**I appreciate your added explanation with regards to screening. However the AREDS trial did not show any benefit for people with early AMD which is why they did a sub-group analysis on people with Intermediate AMD at high risk of progression. Given the positive finding was a post-hoc sub-group analysis, whether AREDS supplements are of any benefit in AMD is controversial. And although AREDS purported to show a reduced risk of progression, there was no significant difference in vision between the groups. Many consider the evidence of benefit too weak to give a firm clinical recommendation on AREDS which is why AREDS supplements are not recommended in the UK by national guidelines (NICE AMD Guidelines 2018). I suggest editing this added paragraph to reflect this uncertainty around AREDS.”

**Our response:** As suggested, the paragraph was edited and described that scientific evidence on the efficacy and safety of use antioxidant vitamin and mineral supplementation is limited based on information from the AREDS study, as described below:

“Currently, there are no medical treatments for early or intermediate AMD, only evidence that antioxidant vitamin and mineral supplementation can delay progression to the advanced stage and the loss of visual acuity in people with signs of the disease. However, the scientific evidence is limited and there are several unanswered questions regarding antioxidant vitamin and mineral supplementation in the prevention of AMD, including at what stage the protective effect may be important and potential interactions with genetic effects and other risk factors for the disease, such as smoking. Furthermore, the safety of antioxidant vitamin and mineral supplementation is controversial[12, 13]”.

“In 2018, the National Institute for Health and Care Excellence (NICE) guideline does not recommend using antioxidant and zinc supplements for AMD. According to this guideline, although the AREDS study showed some beneficial effects of combined antioxidant supplementation in a subgroup of participants, the effects of the individual formula components on AMD progression were unclear and one of the ingredients (beta carotene) was associated with a possible risk of lung cancer among smokers. The guideline also highlights that although the AREDS research group introduced a new formulation that excluded beta carotene in the AREDS2 study, study design limitations related to secondary randomization and no placebo control mean that the effect of this formulation on AMD disease progression remains unknown. A well-conducted randomized trial could therefore provide new evidence on the benefits and risks of individual components of antioxidant supplements [14]”.

**Reviewer #1, comment 2:** “In the final sentence the authors mix screening and clinical monitoring which are different objectives. I suggest that the systematic review evaluated whether AMD screening could be considered effective for identifying patients with early clinical signs of the disease in case treatments for these stages of AMD become available in the future, and identifying patients with advanced AMD for early treatment prior to their self-presentation”.

**Our response:** We appreciate the reviewer's comment. The objective of the study was updated, as described below:

“In this scenario, the present systematic review evaluated whether AMD screening could be considered effective in identifying patients with early clinical signs of the disease should treatments for these stages of AMD become available in the future, as well as those who have progressed to advanced AMD for early treatment prior to their self-presentation.”

**Reviewer #1, comment 3.** Excellent re-write.

**Our response**: Thank you for the comment.

**Reviewer #1, comment 4.** “My point here is that line 38 “(AMD) is an eye disease most prevalent in patients over 50 years old” should read “…is an eye disease that occurs in patients over 50 years old”, and line 60 “that generally affects patients over the age of 50” should read “that affects patients over the age of 50”. Because is only affects patients over 50 years.”

**Our response:** Thank you for the comment. The manuscript was updated with the following information:

- “Age-related macular degeneration (AMD) is an eye disease that occurs in patients over 50 years old.”
- “Age-related macular degeneration (AMD) is a chronic progressive disease that affects patients over the age of 50”.

**Reviewer #1, comment 5 (related to comment 21 from the previous revision).** “I am generally happy with this re-write but in the UK the NHS invites people with diabetes to screening only every 12 months. If more frequent monitoring is required they are referred to ophthalmology clinics. And see comments in Comment 1 above about AREDS”.

**Our response:** Thank you for the comment about screening for diabetic retinopathy in the NHS. The information about screening every 3, 6, 9 or 12 months was updated in the text and included a new reference (NICE Guideline 2018). The manuscript was updated with the following information: “In the United Kingdom, the National Health Service (NHS) invites people with diabetes aged 12 years and over to be screened once a year”.

In the same paragraph, the manuscript was also updated with the following information: “In the case of AMD, studies suggest that vitamin and mineral supplements can delay progression of the disease to the advanced stage, but scientific evidence about efficacy and safety is limited [12, 13]. As such, the NICE guideline does not recommend their use for AMD[14]. Therefore, this systematic review aimed to assess whether AMD screening could be considered effective in identifying patients with early clinical signs of the disease should treatments for these AMD stages available in the future, as well as those who have progressed to advanced AMD for early treatment prior to their self-presentation”.

**Reviewer #3, comment 1:** “Evaluation of revision: authors responded carefully to all comments of both reviewers’ especially reviewer 1. The manuscript was very much improved after corrections and amendments made by the authors”.

- “Despite limited material available, the study provides important information about, in fact, lack of reliable and consistent screening approach to early and intermediate AMD. In the context of new technologies, as mentioned by reviewer 2, the burden of workload for AMD screening might be significantly reduced in the future and implementation of screening protocols based on AI made easily accessible”.
- My minor remark:

a) Please mention preventive laser treatments for dry AMD (micropulse laser, 2-RT laser). Their efficacy is questioned at the moment, but the research is going on. If they have proven really preventive, than early screening would be justified.

**Our response:** Thank you for the comment. The manuscript has been updated with the following information:

“In the future, developing treatments to prevent AMD progression may improve the prognosis of the disease. Research is currently underway on preventive laser treatments for dry AMD, such as micropulse and nanosecond laser techniques [33–35]. The Early Stages of Age-Related Macular Degeneration (LEAD) study, for example, evaluated the safety of subthreshold nanosecond laser treatment in intermediate AMD and its efficacy for slowing progression to late AMD. The results found no significant reduction in the overall rate of progression to late AMD compared with sham treatment in intermediate AMD patients. However, post hoc analyses revealed a potential beneficial effect of subthreshold nanosecond laser (SNL) treatment in eyes without reticular pseudodrusen (RPD) at baseline, and that SNL treatment may increase the rate of progression to late AMD in eyes with RPD at baseline. As such, further studies are needed before recommendations can be made[36].”

**Additional comment**: We would like to inform you that Figure 1 (Study selection flowchart) was updated because the number of articles assessed for eligibility and the number of full articles excluded were revised.
